# Animal-Based Measures to Assess the Welfare of Extensively Managed Ewes

**DOI:** 10.3390/ani8010002

**Published:** 2017-12-24

**Authors:** Carolina Munoz, Angus Campbell, Paul Hemsworth, Rebecca Doyle

**Affiliations:** 1 Animal Welfare Science Centre, The University of Melbourne, North Melbourne, VIC 3051, Australia; phh@unimelb.edu.au (P.H.); rebecca.doyle@unimelb.edu.au (R.D.); 2 Faculty of Veterinary and Agricultural Science, The University of Melbourne, Werribee, VIC 3030, Australia; a.campbell@unimelb.edu.au

**Keywords:** animal-based indicators, animal welfare, kappa statistics, observer agreement, on-farm welfare assessment, sheep

## Abstract

**Simple Summary:**

The aim of this study was to assess the reliability and practicality of 10 animal-based welfare measures for extensively managed ewes, which were derived from the scientific literature, previous welfare protocols and through consultation with veterinarians and animal welfare scientists. Measures were examined on 100 Merino ewes, which were individually identified and repeatedly examined at mid-pregnancy, mid-lactation and weaning. Body condition score, fleece condition, skin lesions, tail length, dag score and lameness are proposed for on-farm use in welfare assessments of extensive sheep production systems. These six welfare measures, which address the main welfare concerns for extensively managed ewes, can be reliably and feasibly measured in the field.

**Abstract:**

The reliability and feasibility of 10 animal-based measures of ewe welfare were examined for use in extensive sheep production systems. Measures were: Body condition score (BCS), rumen fill, fleece cleanliness, fleece condition, skin lesions, tail length, dag score, foot-wall integrity, hoof overgrowth and lameness, and all were examined on 100 Merino ewes (aged 2–4 years) during mid-pregnancy, mid-lactation and weaning by a pool of nine trained observers. The measures of BCS, fleece condition, skin lesions, tail length, dag score and lameness were deemed to be reliable and feasible. All had good observer agreement, as determined by the percentage of agreement, Kendall’s coefficient of concordance (W) and Kappa (k) values. When combined, these nutritional and health measures provide a snapshot of the current welfare status of ewes, as well as evidencing previous or potential welfare issues.

## 1. Introduction

On-farm welfare assessments can be used for immediate or ongoing on-farm monitoring and benchmarking by farmers and veterinarians, and to demonstrate compliance with national and international legal welfare standards and farm assurance schemes [1,2]. For welfare assessments to be effective and acceptable to all key stakeholders (i.e., industry, animals, scientists, consumers and society), they must incorporate welfare measures that are meaningful with respect to animal welfare, provide repeatable outcomes when applied by different observers and practical under farm conditions; that is they must be valid, reliable and feasible [2,3]. Welfare measures can be classified into categories that assess housing and facilities (resource-based measures), farmer policies and management strategies (management-based measures), and direct animal observations (animal-based measures) [4,5]. Animal-based measures often reflect the outcome of resource inputs and management practices, and therefore there is increasing interest to incorporate these measures in welfare assessments, as they provide an integrative and direct measurement of the welfare state of animals [6,7,8]. Some examples of animal-based measures include the assessment of the nutritional state (e.g., body condition score), environmental conditions (e.g., fleece cleanliness) and diseases (e.g., lameness). Welfare measures, however, cannot automatically be extrapolated from one species to another [3].

Welfare challenges differ depending of the species, production system, reproduction cycle and geographical location [9,10,11]. The unique characteristics and welfare challenges of extensive sheep systems highlights the importance of the development of reliable and feasible welfare measures that can be able to detect current welfare problems and risk of future welfare compromise. For instance, the nature of extensive systems, where sheep are managed in large flocks and outdoor all year, makes adequate monitoring, treatment and prevention of diseases more difficult to address. Extensively managed sheep are more exposed to predators and variation in climatic conditions. Variation in food quality and availability during the year leads to changes in body condition, which highlights the importance of measures that can be able to detect these differences. Body condition is widely accepted as a valid and important welfare measure that reflects the nutritional state of sheep [12,13], but discrepancies exist in the literature on the scoring scales and the precision needed (full-unit, half-unit or quarter-unit) to provide meaningful results on the nutritional status of sheep [13,14,15]. In Australia for example, flystrike (cutaneous myiasis) is a main welfare concern, and therefore, it is important to have sensible measures that can identify the risk of this disease. Larsen et al. [16] developed a detail 6-point scale to assess dags, lump matted faecal material hanging from the wool, and although this scoring system has been proved to be effective in assessing the risk of flystrike, it has not been tested for reliability and practicality to be included in welfare assessments. Previously, sheep have received considerably less attention in the development of welfare measures when compared with animals farmed intensively such as pigs, poultry and dairy cattle. Furthermore, most of the research has been conducted in European countries [10,13,15,17,18], where sheep are managed in small flocks and usually in more intensive, indoor-lambing systems [2]. The aim of the present study was to test the reliability and feasibility of some animal based measures for sheep welfare assessment. We hypothesized that some measures previously identified may not be reliable or feasible for sheep managed under extensive farming conditions.

## 2. Materials and Methods

### 2.1. Animals and Management

This study is part of a longitudinal on-farm study that was performed in Victoria, Australia between July and December, 2015 [19]. This study was approved by the University of Melbourne ethics committee (ethical review number 1513562.1). A total of 100 Merino ewes, aged 2–4 years, from a large flock of approximately 3000 breeding ewes were individually identified by a unique ear tag number and repeatedly examined at three-time points: Mid-pregnancy (MP; July), mid-lactation (ML; October) and weaning (WN; December). These periods were selected because they are known to be critical times affecting ewe welfare [15,18]. The ewes were managed under extensive conditions, in a year-round outdoor system, grazing annual/perennial pastures, and managed under commercial conditions. The ewe sample size was selected based on a power calculation assuming 50% prevalence of the trait under observation (the proportion requiring the greatest sample size when observing binomial traits), a 95% confidence interval and precision of ±10%. This number was supported by the AWIN sheep protocol which recommends a sample of 92 animals when the farm size is ≥ 2000 breeding ewes [10].

### 2.2. Animal-Based Welfare Measures

The animal-based measures examined in this study were selected after a review of the relevant literature and consultations with veterinarians and animal welfare scientists. The measures selected were: Body condition score (BCS) [14,20,21,22], rumen fill [23,24], fleece cleanliness [10], fleece condition [10], skin lesions [10], tail length [25,26], dag score [16], foot-wall integrity [27,28,29], hoof overgrowth [27,28,29] and lameness [10]. They were considered valid on the basis that they have been shown to have validity in previous studies, further details are reported in Munoz et al. [19]. The measures selected address main welfare concerns for sheep, covering freedom from hunger, pain, injury or disease. The assessment criteria of the welfare measures are listed in Table 1.

### 2.3. Welfare Assessment of the Ewes

The assessment of the ewes was always conducted between 900 h and 1600 h. To perform the assessment, and for practical reasons, the ewes were managed in four groups of 25 animals. The first nine measures, BCS, rumen fill, fleece cleanliness, fleece condition, skin lesions, tail length, dag score, foot-wall integrity and hoof overgrowth, were assessed in a single-file race within the farm’s regularly-used sheep yards. The ewes were then released from the race (in small groups of 2 to 4 animals) and encouraged to walk to assess lameness. Feasibility of the assessment was measured by timing the assessment at each time-point, evaluating the resources required and the ability to collect these measurements across different farms. The advice of farm consultants, veterinarians and animal welfare scientists was also considered.

### 2.4. Observers and ‘Test Standard Observer’

A pool of nine observers from the University of Melbourne Veterinary and Agricultural Sciences Faculty (details provided in next paragraph) were recruited. Reliability was assessed by evaluating inter- and intra- observer agreement. Observer agreement was assessed in line with previous reliability studies [3,13,15,23]. Briefly, inter- observer agreement and pair agreement was calculated by referencing the score given by each observer against a ‘test standard observer’ (TSO, CM). This approach is commonly used to assess if observers could be trained to apply a specific on-farm welfare assessment protocol and to identify any assessment bias [30,31]. To assess intra-observer reliability, the degree to which measurements taken by the same observer are consistent, all sheep were reassessed by the observers within a 15-day period in MP, and within a 24h period at both ML and WN. In an effort to maintain objectivity, observers did not have access to health or production records of the farm before performing the welfare assessment.

Observer 1 (CM), a veterinarian that developed the list of measures and provided training to all observers was nominated as the TSO. Observer 2 was a research assistant with 25 years of experience in working with sheep and classed as experienced assessor. Observers 4 and 7 were veterinarians classed as mid-experienced observers, and observers 3, 5, 6, 8 and 9 were graduate animal science students, classed as inexperienced observers. From the pool of nine observers, combinations of four observers performed the assessments on each observation period, and the TSO performed the assessment in all the observation periods. This approach was taken because it was difficult to have all the observers in all the farm visits.

Prior to individual assessments, observers were provided with an assessment protocol, containing details of the scoring scales and pictures. In addition, an on-farm training session was provided using 20 ewes at MP, and this training lasted for about 30 min, 25 ewes were used for training at ML, and this training lasted for about 1 h, and 30 ewes were used at WN and this training lasted for about 1 h 30 min. The animals used for training purposes were not included in the analyses. Thereafter, each observer independently evaluated the first nine measures on each sheep. Observers were placed in different locations of the race and were not allowed to exchange their observations. After the assessment in the race, ewes were encouraged to walk to detect lameness. For practical reasons, all the observers assisted with the identification of lame animals. One person was required to move the sheep along the race, another person was required to manipulate the gate at the end of the race and two persons were required to catch the lame sheep for individual identification. Therefore, only intra-assessment agreement was assessed for lameness.

### 2.5. Statistical Analysis

Data analysis was performed using SAS statistical package (Statistical Analysis System, Release 9.4 2012; SAS Institute Inc., Cary, NC, USA). The welfare scoring scales consisted of categorical, ordinal (BCS, fleece cleanliness, fleece condition, skin lesions, dag score, foot-wall integrity, hoof overgrowth and lameness) and binary data (rumen fill and tail length). For ordinal scores, Kendall’s coefficient of concordance (W) was used to assess overall observer agreement [32]. The scale used to assess agreement was as follows: a value of 0 indicates no agreement, from 0.10 to 0.40 poor agreement, 0.41 to 0.70 moderate, 0.71 to 0.90 substantial, 0.91 to 0.99 almost perfect and 1 perfect agreement. Pair-agreement, agreement between individual observers and the TSO, were assessed by the percentage of agreement; Kendall’s W and the weighted kappa statistic (Kw). For binary scores, Fleiss’s Kappa (k) [33] and Cohen’s kappa (k) were used to assess overall observer agreement and pair-agreement respectively. All k results were interpreted according to Landis and Koch [34], therefore values ≤0.40 suggested ‘poor’ agreement, values from 0.41 to 0.60 suggested ‘moderate’ agreement, values ranging from 0.61 to 0.80 suggested ‘substantial’ agreement, and values ≥0.81 suggested ‘almost perfect’ agreement.

In addition, one-way ANOVA analysis was used to examine differences in the ‘time spent assessing the ewes’ between mid-pregnancy, mid-lactation and weaning. Multiple comparisons between means were performed using Fisher’s Least Significant Difference (LSD) test.

## 3. Results

A total of five ewes were lost from the study period, with three ewes dying at lambing (reported dead by the farmer) and two presumed dead, which resulted in different numbers of ewes examined across the three-time points: Mid-pregnancy *n* = 100, mid-lactation *n* = 96 and weaning *n* = 95.

### 3.1. Inter- and Intra-Observer Agreement at Mid-Pregnancy

At mid-pregnancy, there was ‘almost perfect’ overall observer agreement for fleece cleanliness and fleece condition, ‘moderate’ agreement was found for BCS, skin lesions, foot-wall integrity and hoof overgrowth, and ‘poor’ agreement for rumen fill, and tail length (Table 2). In the same way, pair agreement was higher for fleece cleanliness and fleece condition, while BCS, rumen fill and tail length presented the lowest. Overall, the TSO and observer 2, the most experienced observer, had better percentage of pair-agreement for most of the measures compared to the results obtained by the TSO against observers 3 and 4, the less experienced observers. Results for intra-observer agreement are presented in Table 3. Overall, fleece cleanliness and fleece condition were the most repeatable measures. Dag score, foot wall-integrity and hoof overgrowth had moderate repeatability, while BCS and skin lesions had the lowest. Observer 1 and 2 showed the highest levels of repeatability for most of the measures compared to the other observers as determined by W and k values. Lameness was not assessed for intra-observer agreement because all the observers assisted with the identification of lame animals, but showed moderate intra-assessment agreement W = 0.53. According to the observers, rumen fill was the least feasible measures followed by foot-wall integrity and hoof overgrowth. Based on this, rumen fill was not included in the subsequent visits and therefore intra- observer agreement was not assessed.

### 3.2. Inter- and Intra-Observer Agreement at Mid-Lactation

At mid-lactation, ‘substantial’ to ‘almost perfect’ overall agreement was found for fleece cleanliness, fleece condition, BCS and skin lesions. ‘Moderate’ overall agreement was found for dag score, foot-wall integrity and hoof overgrowth, while tail length showed the lowest agreement (Table 4). Similarly, pair agreement was higher for fleece cleanliness, fleece condition and skin lesions. ‘Moderate’ to ‘substantial’ pair agreement was obtained for BCS and dag score while tail length, foot-wall integrity and hoof overgrowth presented the lowest showing from ‘poor’ to ‘substantial’ pair agreement. The results of the intra- observer agreement are present in Table 5. Overall, fleece cleanliness, fleece condition, skin lesions and dag score were the most repeatable measures followed by BCS and tail length, while foot-wall integrity and hoof overgrowth presented the lowest levels of repeatability. The TSO showed the highest levels of repeatability for most the measures, and her repeatability increased at mid-lactation when compared to mid-pregnancy, particularly for the measures BCS and dag score that increased from ‘moderate/poor’ to ‘substantial/moderate’ agreement. The intra-assessment agreement of lameness increased to ‘substantial’ W = 0.79.

### 3.3. Inter- and Intra-Observer Agreement at Weaning

At weaning, most of the welfare measures presented from ‘moderate’ to ‘almost perfect’ overall agreement (Table 6). ‘Almost perfect’ pair agreement was obtained for fleece cleanliness, fleece condition and skin lesions. Body condition score, dag score and hoof overgrowth ranged from ‘poor-moderate’ to ‘almost perfect’ pair agreement. Foot-wall integrity and tail length had the lowest pair agreement, however k values for tail length ranged from 0.22 (‘poor’) to 1.00 (‘almost perfect’). The intra-observer agreement results are presented in Table 7. The most repeatable measures at weaning were fleece cleanliness, fleece condition, skin lesion and BCS followed by dag score and tail length. The least repeatable measures were foot-wall integrity and hoof overgrowth. The intra-assessment agreement of lameness also increased significantly at weaning showing substantial levels of repeatability W = 0.86. The TSO showed the highest levels of repeatability, and her repeatability increased significantly, particularly for BCS, dag score and tail length which increased from ‘substantial/moderate’ at mid-lactation to ‘substantial/almost perfect’ at weaning.

**Figure 1:**
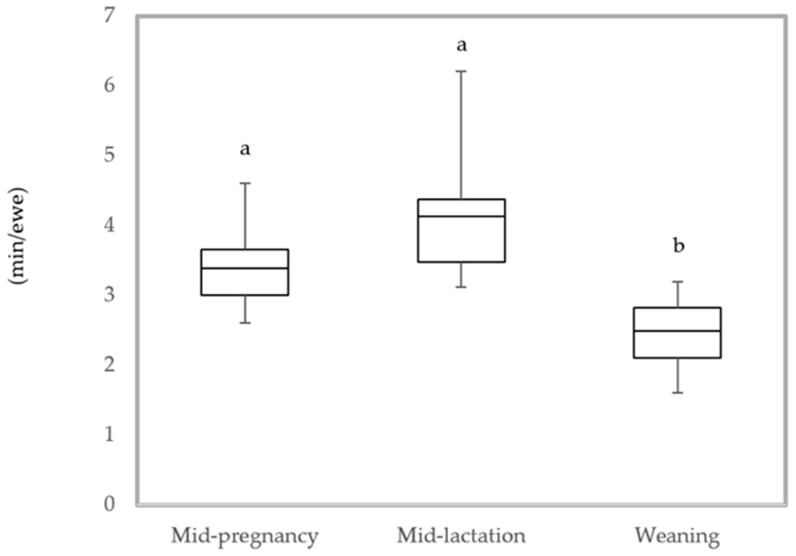
Time spent assessing the ewes (min/ewe) at mid-pregnancy, mid-lactation and weaning. Different letters indicate statistical difference (*p* < 0.05).

The welfare assessment of the ewes using 10 animal-based measures took from 4 to 6 h. No differences in the time spent assessing the ewes were found between mid-pregnancy and mid-lactation, means were 3.4 min/ewe (SD ± 0.63) and 4.1 min/ewe (SD ± 1.03) respectively. However, the time spent performing the assessment significantly decreased (*p* = 0.001) at weaning to 2.5 min/ewe (SD ± 0.56), Figure 1.

## 4. Discussion

This study assessed the reliability and feasibility of 10 animal-based welfare measures for extensively managed ewes. Body condition score, fleece condition, skin lesions, tail length, dag score and lameness are proposed for on-farm use in welfare assessments of extensive sheep production systems. These six valid measures address the main welfare concerns for ewes, and they are reliable and feasible. When combined, they provide an overview of the nutritional, health and welfare state of the ewes as well as evidencing previous or potential welfare concerns.

### 4.1. Reliability of the Animal-Based Welfare Measures

High inter- and intra- observer agreements, from ‘substantial/moderate’ to ‘substantial/almost perfect’ agreements, were found for BCS, fleece cleanliness, fleece condition, skin lesions, tail length, dag score and lameness. In the present study, BCS was the measure that increased the most, the inter-observer agreement and the intra-agreement of the TSO increased from ‘moderate’ at mid-pregnancy to ‘almost perfect’ at weaning. Body condition is widely accepted as a valid and important welfare measure that reflects the nutritional state of sheep [13,14]. Results in the present study suggests that a quarter-point scale is reliable, but that operators require sufficient training and experience to achieve high agreement in this measurement [27,35,36]. In this study, the experienced observers (TSO, observers 2 and 7) showed the highest agreement and repeatability for this measure. The increased training sessions and the clarification of the descriptive terms used may have help to achieved ‘almost perfect’ inter- and intra- observer agreement at the end of the study. Although individual differences, observer expertise and differences in intervals of reassessment (15-day period at MP vs. 24 h at ML and WN) may have influenced in the levels of agreement obtained, there is evidence that the level of observer agreement increases significantly when sufficient training is provided [13,18].

Rumen fill, foot-wall integrity and hoof overgrowth were the measures with lower agreement in this study. This is likely the result of difficulties associated with assessing these measures, e.g., presence of fleece and the fact that ewes often moved backwards and forwards along the race, which particularly affected how easily foot-wall integrity could be assessed. In addition, the scoring scales and the descriptive terms used for foot-wall integrity may have affected the levels of observer agreement. Simplifications of the scoring scales as well as clarification of the description terms may provide higher agreement and may be more useful for future on-farm assessments.

The performance of each welfare measure was evaluated in agreement with previous reliability studies [13,18,23,27]. Percentage of agreement was used as it provides an easy illustration of observer agreement. However, as this method does not estimate the amount of agreement that could occur by chance, Kendall’s coefficient of concordance (W) and Kappa (k) were selected to statistically assess the inter and intra-observer agreement of ordinal and binominal measures. Care is needed however when interpreting k values, because they are affected by the prevalence of the condition under consideration. Populations with few animals presenting the condition of interest will provide very low values of k that may not necessarily reflect low levels of observer agreement [37]. In the present study, the length of the tail was a simple binominal scale and presented high percentage of agreement across the three-time points examined (MP: 71–86%; ML: 85–97%; WN: 96–100%). However, k values were consistently low; from 0.28 to 0.39 at MP, from −0.01 to 0.56 at ML and from 0.37 to 1.00 at WN. Discrepancies between the percentage of agreement and k values may be a consequence of the low number of animals that had adequate tail length in this study (*n* = 8, as determined by the TSO, while 92% *n* = 87 had short-docked tails at weaning), and may not necessarily mean low inter- observer agreement. It is possible that higher k values would have been achieved if more animals in this study had adequate tail length. Similar difficulties in the interpretation of k values have been reported in previous studies [23,37,38]. Other factors that need to be considered when evaluating reliability is intervals of reassessments. In the present study, low intra observer reliability at mid-pregnancy cannot be completely attributed to lack of consistency of the observers, as the length of the reassessment at this stage (15-day period) may have affected the levels of intra- observer agreement of dag score, skin lesions, foot-wall integrity and hoof overgrowth.

Overall, there is wide variation in the scientific literature on how reliability of welfare measures is assessed. Currently, there is no agreement on the number of animals, number of observers or the methodology that should be used. For instance, a reliability study in lambs used four observers to assess 966 lambs [23], a study of welfare assessment for adult sheep used two observers and 360 ewes [15], and studies assessing reliability on locomotion scoring in various species have used five observers and 83 cows [39], three observers and 30 video clips of sheep [40], and three observers and 80 photographs and videos of foot-rot lesions in sheep [27]. The sample size selected in the present study was based on a power calculation and recommendations by the AWIN sheep protocol [10], and the fact that the performance of the measures was tested on-farm during different stages of production of sheep further supports their reliability and applicability under farm conditions.

### 4.2. Feasibility of the Animal-Based Welfare Measures

Welfare measures need to be practical if they are to be valuable. Sheep farms in Australia can commonly have 12,000 animals, and they are usually managed by a single person [9,41]. This, highlights the need for feasible measures that can be taken in short periods of time with low need of resources and personnel as time and labor force are limited in extensive sheep systems. When assessing the feasibility of the measures of this study a variety of factors were considered such as time spent in the assessment, resources required and the ability to collect these measurements across different farms. Feasibility was assessed for a third party to perform the assessment, not a farmer. Generally, the measures tested proved to be feasible, requiring on average 2.5 min to assess an individual ewe at weaning. The significant decrease in the time spent in the assessment at weaning might have been influenced by individual differences of the observers, and familiarization with the scoring scales and assessment protocol. Although no differences were found in the time spent assessing the ewes between mid-pregnancy and mid-lactation. Lactation was considered the least practical period due to the presence of lambs, which made sheep handling difficult during the assessment. This needs to be considered when deciding for key times to perform on-farm welfare assessments.

The most feasible measures were found to be BCS, fleece cleanliness, fleece condition, skin lesions, tail length, dag score and lameness. Clear advantages of these measures in terms of practicality are that no measures required specialized equipment; the only infrastructure required is a raceway, which is a common facility on sheep farms, and other than the labor required to bring the sheep into the yards, they do not interrupt farm management practices. It should also be considered that most farmers visually monitor their sheep in the paddock, rather than gathering them into the yards. In this context, it has been shown that some of these measures, e.g., thin body condition, lameness and dags can be examined from the distance during key stages of the production cycle [2,42] with minimal interference with farm work. Thus, the measures selected may be considered more acceptable by producers. Foot-wall integrity and hoof overgrowth on the other hand, were found less practical as they were time-consuming and they were not easy to assess as ewes often moved backwards and forwards. Additionally, their implementation across farms is limited as they should be assessed in races with no covered walls alongside.

### 4.3. Recommended Measures for On-Farm Welfare Assessment of Extensively Managed Ewes

This research is important because it identified measurements the are suitable for use under commercial conditions [43]. The validity of these measures reported in Munoz et al. [19], plus their reliability and feasibility examined in this study indicate that these six animal-based measures; BCS, fleece condition, skin lesions, tail length, dag score and lameness are appropriate/recommended to include in welfare protocols for ewes managed extensively, particularly in Australia. When these measures are combined, they provide a snapshot of the current welfare status of ewes, as well as providing evidence of past or potential welfare risks. For example, combining a decline in BCS, poor fleece condition and high dag score helps to identify that the welfare of that animal is compromised, while also facilitating the identification of the problem and the appropriate treatment. These measures address important welfare issues identified by producers, industry, specialist and general public [10,38,44].

Fleece cleanliness, although repeatable and feasible, might not be meaningful for extensive systems. Fleece cleanliness has previously been proposed as an important welfare measure for sheep, as it can provide information about the quality of the environment [10,15,18,23,45]. However, this measure is more valuable for intensive indoor lambing systems where is important to assess the cleanliness of the floor/bedding and how the animal is coping with this environment. Rumen fill, foot-wall integrity and hoof overgrowth were discarded based on poor reliability and feasibility. Rumen fill has been identified as a relevant animal-based measure for sheep and lambs as provides short-term information of food access [38]. In the present study, rumen fill was difficult to assess and this was reflected in the poor levels of agreement achieved. The presence of the fleece was the main factor affecting the levels of inter-observer agreement. Similar results have been obtained in a previous study on lambs where only ‘moderate’ inter-observer agreement was obtained [2]. In view of the difficulties of assessing rumen fill in ewes that are not in short wool and its limitations in assessing sheep welfare, the measure was excluded. Foot-wall integrity and hoof overgrowth showed poor repeatability and feasibility to be implemented across different farms. It should also be considered that broader measures, such as lameness, may be more relevant to assess ewe welfare than foot-wall integrity and hoof overgrowth.

Besides the importance of discriminating which welfare measures would be more suitable for extensive conditions, it is also important to identify alternatives that could be used to measure on-farm welfare in sheep. For instance, limited research has been done to develop practical assessments of fear of humans in sheep, and studies on this topic vary in methodology and performance [46]. The majority of this research has been focused on intensively managed sheep [15,47,48,49], and usually under experimental conditions [41,47,50]. Further work is needed to validate a practical on-farm assessment of fear of humans that could be applied to extensive systems. Recent studies by Hazard et al. [51,52] have investigated several behavioral traits in sheep that could be used to validate the assessment of fear of human in extensive farming conditions. Additionally, limited work has been done to develop practical on-farm assessments for clinical and sub-clinical mastitis [10]. Udder examination and collection of milk samples to perform an on-farm test (e.g., California mastitis test) is time-consuming and labor intensive, which make these assessments less appealing for on-farm use. Further studies in the development of practical welfare assessments should consider the incorporation of new technologies for practical assessment of mastitis and to track grazing behavior and sheep movement to detect sick/lame animals. Finally, it should be considered that extensive systems are characterized by seasonal variation in both, climate and food availability, which results in seasonal variation in the welfare status of sheep [18]. Welfare measures therefore must be able to detect variation in the welfare status of ewes over main risk periods of the production cycle [18], as well as be sensible to identify differences between farms. Further research into the development of welfare assessment for extensive systems should assess both seasonal variation of the measures selected and their ability to detect differences between farms as only one property was examined in the present study.

## 5. Conclusions

The results obtained in the present study suggest that BCS, fleece condition, skin lesions, tail length, dag score and lameness are reliable and feasible measures that can be included in welfare protocols for extensive sheep production systems. The high levels of inter- and intra- agreement found for these measures also suggests that the scoring scales and the descriptive terms used are reliable. When these measures are used in combination with resource-based and management-based measures they can be used to address welfare compromise. Lactation was considered the least practical period due to the presence of lambs, which needs to be considered when deciding for key times to perform on-farm welfare assessments. Further research examining the ability of these measures to detect seasonal variation and between-farm differences will provide further evidence of their effectiveness in assessing the welfare condition of ewes managed extensively.

## Figures and Tables

**Table 1:** Animal-based welfare measures used to assess the welfare of extensively managed ewes.

Measure	Assessment Criterion
Body condition score	Scored on a 5 point scale from 1 (thin) to 5 (obese), using a quarter-unit precision [14,21]. Sheep were assessed by palpation of the backbone, muscle and short ribs [20,22].
Rumen fill	Scored on a 2 point scale: (0) If the animal’s left-hand side is not sunken/or is convex between the hip bone and the ribs and (1) if the animals’ left-hand side is deeply sunken between the hip bone and the ribs [23,24].
Fleece cleanliness	Scored on a 4 point scale: (0) Clean and dry (1) dry with slight mud/dirt (2) Wet with some areas contaminated by mud or dung (3) Filthy, very wet and coated in mud or dung [10].
Fleece condition	Scored on a 3 point scale: (0) Good fleece condition, when parted, the fleece has no scurf or lumpiness or signs of ectoparasites (1) some fleece loss, small shed or bald patches of no more than 10 cm diameter. When parted, the fleece may have some lumpiness or scurf, little evidence of ectoparasites, and (2) significant fleece loss with bald patches of greater than 10 cm in diameter, clear evidence of ectoparasites [10].
Skin lesions	Assessed by recording number, location and severity of the skin lesions. Lesions were classified as cuts, open wounds, old wounds or scars and abscesses [10].
Tail length	Scored on a 2 point scale: (0) The tip of the vulva is covered by the tail when held down (1) the tail is over-shortened or almost not present, or if the vulva and anus cannot be covered [25,26].
Dag score	Scored on a 6 point scale: (0) No evidence of fecal soiling, (1) very light soiling on the breech area, (2) moderate dag on the breech area extending ventrally, (3) severe dag predominantly on the breech area, extending ventrally and dorsally over the tail some soiling and dag around anus, (4) excessive dag on the breech area and on the hind legs (5) Very severe dag on the breech area and on the hind legs or below the level of the hocks [16].
Foot-wall integrity	Scored on a 4 point scale: (0) An undamaged wall, (1) 25% or less damaged wall (2) moderately damaged wall (from 25% to 75%), (3) severely damaged wall (>75%) [27,28,29].
Hoof overgrowth	Scored on a 3 point scale: (0) Appropriate length of the hoof and perfect shape of the wall area, (1) moderately misshapen/overgrowth, (2) a severely misshapen/overgrowth [27,28,29]
Lameness	Scored on a 4 point scale: (0) Not lame, (1) clear shortening of stride with obvious head nodding or flicking as the affected limb touches the floor, (2) clear shortening of stride with obvious head nodding and not weight-bearing on affected limb whilst moving, (3) reluctant to stand or move [10].

**Table 2:** Overall observer agreement (OA), percentage of agreement (%) and pair agreement at mid-pregnancy.

Measures	OA (W)	Pair Agreement				
		Observer Identity	%	Kendall’s (W)	Weighted Kappa (95% CI)	Interpretation (W/Kw)
BCS		2	30	0.78	0.38 (0.28–0.54)	Substantial/poor
	0.60	3	24	0.74	0.30 (0.16–0.38)	Substantial/poor
		4	23	0.71	0.25 (0.09–0.33)	Substantial/poor
Rumen fill		2	73	n/a	0.12 ** (−0.11–0.32)	Poor
	0.14 *	3	70	n/a	0.13 ** (−0.08–0.34)	Poor
		4	75	n/a	0.31 ** (0.09–0.47)	Poor
Fleece cleanliness		2	100	1.00	1.00 (1.00–1.00)	Perfect agreement
	1.00	3	100	1.00	1.00 (1.00–1.00)	Perfect agreement
		4	100	1.00	1.00 (1.00–1.00)	Perfect agreement
Fleece condition		2	100	1.00	1.00 (1.00–1.00)	Perfect agreement
	1.00	3	100	1.00	1.00 (1.00–1.00)	Perfect agreement
		4	100	1.00	1.00 (1.00–1.00)	Perfect agreement
Skin lesions		2	98	0.50	0.66 (0.65–0.67)	Moderate
	0.41	3	99	0.83	0.66 (0.04–1.00)	Substantial/moderate
		4	99	0.69	0.39 (−0.17–0.93)	Moderate/poor
Tail length		2	86	n/a	0.38 ** (0.14–0.64)	Poor
	0.35 *	3	77	n/a	0.28 ** (0.10–0.45)	Poor
		4	71	n/a	0.28 ** (0.10–0.45)	Poor
Dag score		2	77	0.81	0.59 (0.43–0.72)	Substantial/moderate
	0.70	3	76	0.77	0.52 (0.37–0.66)	Substantial/moderate
		4	74	0.83	0.62 (0.48–0.76)	Substantial/moderate
Foot-wall integrity		2	90	0.68	0.47 (−0.15–1.00)	Moderate
	0.44	3	97	0.50	0.21 (−0.15–0.57)	Moderate/poor
		4	95	0.57	0.55 (0.20–0.90)	Moderate
Hoof overgrowth	0.65	2	91	0.84	0.66 (0.51–0.80)	Substantial/moderate
		3	79	0.75	0.50 (0.33–0.68)	Substantial/moderate
		4	66	0.63	0.43 (0.27–0.60)	Moderate

* Inter-observer agreement for nominal measures determined by Fleiss’s Kappa (k); ** Pair-agreement with the test standard observer for nominal measures determined by Cohen’s Kappa (k).

**Table 3:** Intra-observer agreement at mid-pregnancy.

Measures	Observer Identity	W	Kw	Interpretation (W/Kw)
BCS	1	0.68	0.22	Moderate/poor
	2	0.80	0.31	Substantial/poor
	3	0.61	0.10	Moderate/poor
	4	0.68	0.20	Moderate/poor
Fleece cleanliness	1	1.00	1.00	Perfect agreement
	2	1.00	1.00	Perfect agreement
	3	1.00	1.00	Perfect agreement
	4	1.00	1.00	Perfect agreement
Fleece Condition	1	1.00	1.00	Perfect agreement
	2	1.00	1.00	Perfect agreement
	3	1.00	1.00	Perfect agreement
	4	1.00	1.00	Perfect agreement
Skin lesions	1	0.49	0.48	Moderate
	2	1.00	1.00 **	Perfect agreement
	3	0.49	−0.01 **	Moderate/poor
	4	0.48	0.56 **	Moderate
Dag score	1	0.63	0.37	Moderate/poor
	2	0.64	0.39	Moderate/poor
	3	0.63	0.43	Moderate
	4	0.6	0.45	Moderate
Foot-wall Integrity	1	0.64	0.37	Moderate/poor
	2	0.58	0.39	Moderate/poor
	3	0.50	0.43	Moderate
	4	0.59	0.45	Moderate
Hoof overgrowth	1	0.79	0.57	Substantial/moderate
	2	0.78	0.50	Substantial/moderate
	3	0.83	0.59	Substantial/moderate
	4	0.72	0.44	Substantial/moderate

** Pair-agreement for nominal measures determined by Cohen’s Kappa (k). Intra-observer agreement was done within a 15-day period.

**Table 4:** Overall observer agreement (OA), percentage of agreement (%) and pair agreement at mid-lactation.

	Pair Agreement					
Measures	OA (W)	Observer Identity	%	Kendal’s (W)	Weighted Kappa (95% CI)	Interpretation (W/Kw)
BCS	0.74	2	48	0.85	0.55 (0.53–0.76)	Substantial/moderate
		5	23	0.83	0.41 (0.29–0.50)	Substantial/moderate
		6	26	0.85	0.45 (0.36–0.55)	Substantial/moderate
Fleece cleanliness	1.00	2	100	1.00	1.00 (1.00–1.00)	Perfect agreement
		5	100	1.00	1.00 (1.00–1.00)	Perfect agreement
		6	100	1.00	1.00 (1.00–1.00)	Perfect agreement
Fleece condition	0.75	2	98	0.83	0.66 (0.04–1.00)	Substantial
		5	100	1.00	1.00 (1.00–1.00)	Perfect agreement
		6	100	1.00	1.00 (1.00–1.00)	Perfect agreement
Skin lesions	0.99	2	100	1.00	1.00 (1.00–1.00)	Perfect agreement
		5	100	1.00	1.00 (1.00–1.00)	Perfect agreement
		6	100	1.00	1.00 (1.00–1.00)	Perfect agreement
Tail length	0.18 *	2	97	n/a	−0.01 ** (−0.03–0.00)	Poor
		5	97	n/a	−0.01 ** (−0.03–0.01)	Poor
		6	97	n/a	−0.01 ** (−0.03–0.01)	Poor
Dag score	0.69	2	65	0.87	0.62 (0.49–0.76)	Substantial
		5	64	0.85	0.47 (0.34–0.61)	Substantial/moderate
		6	63	0.77	0.40 (0.24–0.55)	Substantial/poor
Foot-wall integrity	0.45	2	96	0.65	0.75 (0.44–1.00)	Moderate/substantial
		5	86	0.60	0.32 (−0.18–0.81)	Moderate/poor
		6	94	0.47	0.53 (−0.02–1.00)	Moderate
Hoof overgrowth	0.56	2	66	0.80	0.48 (0.31–0.64)	Substantial/moderate
		5	66	0.75	0.30 (0.22–0.48)	Substantial/poor
		6	40	0.79	0.27 (0.10–0.36)	Substantial/poor

* Inter-observer agreement for nominal measures determined by Fleiss’s Kappa (k); ** Pair-agreement with the test standard observer for nominal measures determined by Cohen’s Kappa (k).

**Table 5:** Intra-observer agreement at mid-lactation.

Measures	Observer Identity	W	Kw	Interpretation (W/Kw)
BCS	1	0.87	0.57	Substantial/moderate
	2	0.79	0.49	Substantial/moderate
	5	0.76	0.37	Substantial/poor
	6	0.62	0.22	Moderate/poor
Fleece cleanliness	1	1.00	1.00	Perfect agreement
	2	1.00	1.00	Perfect agreement
	5	1.00	1.00	Perfect agreement
	6	1.00	1.00	Perfect agreement
Fleece Condition	1	1.00	1.00	Perfect agreement
	2	0.83	0.89	Substantial
	5	1.00	1.00	Perfect agreement
	6	0.75	0.78	Substantial
Skin lesions	1	0.83	0.66 **	Substantial
	2	0.75	0.49 **	Substantial/moderate
	5	0.83	0.66 **	Substantial
	6	0.69	0.49 **	Moderate
Tail length	1	n/a	0.58 **	Moderate
	2	n/a	1.00 **	Perfect agreement
	5	n/a	0.50 **	Moderate
	6	n/a	0.02 **	Poor
Dag score	1	0.87	0.60	Substantial/moderate
	2	0.85	0.61	Substantial
	5	0.90	0.67	Substantial
	6	0.70	0.32	Substantial/poor
Foot-wall Integrity	1	0.73	0.65	Substantial
	2	0.62	0.31	Moderate/poor
	5	0.54	0.21	Moderate/poor
	6	0.48	0.30	Moderate/poor
Hoof overgrowth	1	0.77	0.49	Substantial/moderate
	2	0.74	0.39	Substantial/poor
	5	0.79	0.55	Substantial/moderate
	6	0.60	0.13	Moderate/poor

** Pair-agreement for nominal measures determined by Cohen’s Kappa (k). Intra-observer agreement was done within a 24 h period.

**Table 6:** Overall observer agreement (OA), percentage of agreement (%) and pair agreement at weaning.

Measures	OA (W)	Pair Agreement				
		Observer Identity	%	Kendall’s (W)	Weighted Kappa (95% CI)	Interpretation (W/Kw)
BCS	0.80	7	38	0.90	0.63 (0.54–0.72)	Almost perfect/substantial
		8	39	0.88	0.59 (0.48–0.70)	Substantial/moderate
		9	31	0.86	0.39 (0.27–0.50)	Substantial/poor
Fleece cleanliness	1.00	7	100	1.00	1.00 (1.00–1.00)	Perfect agreement
		8	100	1.00	1.00 (1.00–1.00)	Perfect agreement
		9	100	1.00	1.00 (1.00–1.00)	Perfect agreement
Fleece condition	0.93	7	90	0.88	0.60 (0.41–0.80)	Substantial/moderate
		8	99	0.96	0.88 (0.73–1.00)	Almost perfect
		9	99	0.92	0.88 (0.71–1.00)	Almost perfect
Skin lesions	0.96	7	99	0.96	0.92 (0.76–1.00)	Almost perfect
		8	100	1.00	1.00 (1.00–1.00)	Perfect agreement
		9	99	0.96	0.92 (0.76-1.00)	Almost perfect
Tail length	0.49 *	7	97	n/a	0.65 ** (0.29–1.00)	Substantial
		8	100	n/a	1.00 ** (1.00–1.00)	Perfect agreement
		9	94	n/a	0.22 ** (−0.19–0.62)	Poor
Dag score	0.68	7	79	0.75	0.35 (0.22–0.48)	Substantial/poor
		8	90	0.83	0.53 (0.40–0.65)	Substantial/moderate
		9	87	0.83	0.52 (0.39–0.64)	Substantial/moderate
Foot-wall integrity	0.52	7	92	0.63	0.43 (0.07–0.79)	Substantial/moderate
		8	92	0.83	0.64 (0.36–0.93)	Substantial
		9	93	0.68	0.37 (−0.03–0.76)	Substantial/poor
Hoof overgrowth	0.61	7	76	0.70	0.52 (0.37–0.67)	Substantial/moderate
		8	75	0.71	0.51 (0.36–0.65)	Substantial/moderate
		9	73	0.77	0.48 (0.32–0.64)	Substantial/moderate

* Inter-observer agreement for nominal measures determined by Fleiss’s Kappa (k); ** Pair-agreement with the test standard observer for nominal measures determined by Cohen’s Kappa (k).

**Table 7:** Intra-observer agreement at weaning.

Measures	Observer Identity	W	Kw	Interpretation (W/Kw)
BCS	1	0.90	0.64	Almost perfect/substantial
	7	0.87	0.56	Substantial/moderate
	8	0.87	0.58	Substantial/moderate
	9	0.85	0.59	Substantial/moderate
Fleece cleanliness	1	1.00	1.00	Perfect agreement
	7	1.00	1.00	Perfect agreement
	8	1.00	1.00	Perfect agreement
	9	1.00	1.00	Perfect agreement
Fleece Condition	1	0.92	0.88	Almost perfect
	7	0.91	0.65	Almost perfect/substantial
	8	0.86	0.74	Substantial
	9	0.77	0.69	Substantial
Skin lesions	1	0.86	0.82	Substantial/almost perfect
	7	0.95	0.90	Almost perfect
	8	0.86	0.71	Substantial
	9	0.90	0.65	Almost perfect/substantial
Tail length	1	n/a	0.80 **	Substantial
	7	n/a	0.54 **	Moderate
	8	n/a	0.80 **	Substantial
	9	n/a	0.18 **	Poor
Dag score	1	0.79	0.61	Substantial
	7	0.70	0.37	Substantial/poor
	8	0.65	0.41	Substantial/moderate
	9	0.76	0.48	Substantial/moderate
Foot-wall Integrity	1	0.79	0.58	Substantial/moderate
	7	0.48	0.23	Moderate/poor
	8	0.66	0.48	Moderate
	9	0.70	0.34	Substantial/poor
Hoof overgrowth	1	0.77	0.54	Substantial/moderate
	7	0.75	0.49	Substantial/moderate
	8	0.62	0.32	Moderate/poor
	9	0.63	0.33	Moderate/poor

** Pair-agreement for nominal measures determined by Cohen’s Kappa (k). Intra-observer agreement was done within a 24 h period.
